# Association between dioxin and cancer incidence and mortality: a meta-analysis

**DOI:** 10.1038/srep38012

**Published:** 2016-11-29

**Authors:** Jinming Xu, Yao Ye, Fang Huang, Hanwen Chen, Han Wu, Jian Huang, Jian Hu, Dajing Xia, Yihua Wu

**Affiliations:** 1 Department of Toxicology, Zhejiang University School of Public Health, Hangzhou, 310058, China; 2 Second Affiliated Hospital, Zhejiang University School of Medicine, Hangzhou, 310009, China; 3 Department of Oncology, Second Affiliated Hospital, Zhejiang University School of Medicine, Hangzhou, 310009, China; 4 Department of Thoracic Surgery, The first Affiliated Hospital, Zhejiang University School of Medicine, Hangzhou, 310003, China

## Abstract

The objective of the present study was to systematically assess the association between dioxin/2,3,7,8-tetrachlorodibenzo-p-dioxin (TCDD) and cancer incidence and mortality. Systematic literature searches were conducted until July 2015 in Pubmed, Embase and Cochrane library to identify relevant studies. A random-effects model was applied to estimate the pooled odds ratio (OR), risk ratio (RR), standard incidence ratio (SIR) or standard mortality ratio (SMR) for cancer incidence or mortality. In addition, dose-response, meta-regression, subgroup, and publication bias analyses were conducted. Thirty-one studies involving 29,605 cancer cases and 3,478,748 participants were included. Higher external exposure level of TCDD was significantly associated with all cancer mortality (pooled SMR = 1.09, 95% CI: 1.01–1.19, p = 0.04), but not all cancer incidence (pooled RR = 1.01, 95% CI: 0.97–1.06, p = 0.49). Higher blood level of TCDD was both significantly associated with all cancer incidence (pooled RR = 1.57, 95% CI: 1.21–2.04, p = 0.001) and all cancer mortality (pooled SMR = 1.45, 95% CI: 1.25–1.69, p < 0.001). Subgroup analysis suggested that higher external exposure and blood level of TCDD were both significantly associated with the mortality caused by non-Hodgkin’s lymphoma. In conclusion, external exposure and blood level of TCDD were both significantly associated with all cancer mortality, especially for non-Hodgkin’s lymphoma.

Cancer constitutes an enormous burden on society in more and less economically developed countries. An estimated 14.1 million new cancer cases and 8.2 million cancer deaths occurred in 2012 worldwide^1^. As one of the important established risk factors for cancer, environmental carcinogen like dioxin might contribute to its increasing prevalence^2^,^3^. 2,3,7,8-tetrachlorodibenzo-p-dioxin (TCDD or dioxin) is the most toxic halogenated aromatic hydrocarbon^4^, which is a widespread the environmental contaminant released by various sources of combustion, incineration, and chemical manufacturing^5^,^6^. This compound is extremely stable and thus accumulates in the food chain with a half-life of 7–9 years in humans^7^,^8^. In 1997, the International Agency for Research on Cancer (IARC) has classified it as a known human carcinogen (group 1) on the basis of animal studies and mechanistic information, but the epidemiology data was limited^2^. In 2012, the IARC illustrated the associations between TCDD and human cancers according to many observational studies^3^, but these issues were not systematically reviewed and quantified by a meta-analysis. Molecular studies has proven that TCDD is a potent a carcinogen which could disrupt multiple endocrine pathways via aryl-hydrocarbon receptors (AhR) widely present in animals and humans^2^,^8^,^9^.

As mentioned above, many epidemiological cohort studies and case-control studies have evaluated the association between TCDD/dioxin and cancer incidence and mortality^10^,^11^,^12^,^13^,^14^,^15^,^16^,^17^,^18^,^19^,^20^,^21^,^22^,^23^,^24^,^25^,^26^,^27^,^28^,^29^,^30^,^31^,^32^,^33^,^34^,^35^,^36^,^37^,^38^,^39^,^40^, but the results remained inconsistent. In addition, two previous meta-analyses reported the association between TCDD exposure and prostate cancer^41^ and lung cancer^42^, while another^43^ reported the dose-response relationship for blood level of TCDD and cancer mortality based on 3 cohort studies. However, to date, no study has systematically analyzed the association between external exposure or blood level of TCDD and all cancer incidence and mortality. Thus, the aim of this study was to provide a systematically quantitative assessment of the association from an epidemiological point of view, and fill in gaps in the IARC deficiencies on this issue.

## Materials and Methods

### Data sources, search strategy and selection criteria

Systematic literature searches were conducted in PUBMED, EMBASE and Cochrane library (up to July 2015) to identify eligible studies. The following terms were used in the search procedure: (“dioxin” or “TCDD” or “Tetrachlorodibenzodioxin” or “2,3,7,8-Tetrachlorodibenzo-p-dioxin” or “Tetrachlorodibenzo-p-dioxin”) AND (“cancer” or “tumor” or “tumour” or “carcinoma” or “neoplasm” or “sarcoma” or “melanoma” or “malignancy” or “leukemia” or “leukeamia” or “myeloma” or “lymphoma” or “adenoma”). Reports cited the references identified in this systematic review and relevant reviews were also searched to include potentially missed studies. Titles and abstracts were first scanned, and then full articles of potential eligible studies were reviewed. The retrieved studies were carefully examined to exclude potential duplicates or overlapping data. For duplicate reports, the ones with larger sample size, longer follow-up time and/or more detailed information were selected. This meta-analysis was designed, conducted and reported according to PRISMA and MOOSE statements^44^,^45^.

Studies were eligible for inclusion if all the following criteria were fulfilled: (1) prospective or retrospective cohort studies and case-control studies evaluated the association between dioxin/TCDD and cancer incidence and mortality; (2) the odds ratio (OR), risk ratio (RR), standard incidence ratio (SIR) or standard mortality ratio (SMR) estimates and their 95% confidence intervals (95% CI) were given or sufficient data were available for evaluation; (3) articles as full papers in English were evaluated for eligibility. Studies reported the association between Agent Orange/herbicides and cancer incidence and mortality were excluded because the limitation of precise data on TCDD. For studies conducted in the same population, the criteria priority was established according to (1) whether the detailed information of different cancer subtypes and dioxin exposure level was provided or studies with a larger sample size and (2) the publication time. Reviews, meeting abstracts, notes, comments, editorials, and case reports were excluded because of the limited data.

### Data extraction and quality assessment

Data extraction was carried out independently by two investigators (Drs. Xu JM and Ye Y). Discrepancies were resolved by a third investigator. The endpoints of this analysis were all cancer incidence and mortality as most of the included studies adopted, as well as site/type-specific cancers. The following information was extracted from each study: authors, year of publication, country of each study, study period, population characteristics (sample size, gender and age), and cancer subtypes. ORs (RRs, SIRs or SMRs) reflected the greatest degree of control for potential confounders were adopted in this meta-analysis. The quality of each study was assessed according to NEWCASTLE-OTTAWA quality assessment^46^. The total score ranges from 0 to 9, and a higher score indicates higher quality. Sensitivity analyses are further conducted according to the quality assessment results to explore the source of heterogeneity.

### Data synthesis and statistical analysis

The primary meta-analyses were conducted to assess the association between external exposure and blood level of TCDD and all cancer incidence and mortality. Heterogeneity between individual studies was assessed by the chi-square test and I^2^ test; P ≤ 0.10 and/or I^2^ > 50% indicates significant heterogeneity^47^. Summary ORs (RRs, SIRs or SMRs) and 95% CI were calculated using a random-effects model. The significance of the pooled ORs (RRs, SIRs or SMRs) were determined by Z test (p < 0.05 was considered to be significant). Studies that reported results of a specific type of cancer but no data on all cancer were not pooled for all cancer analysis. Subgroup analyses were applied to explore source of heterogeneity and to evaluate potential effect of modification of variables including cancer subtype, exposure way and TCDD exposure reference category. In order to avoid bias and make the analysis more accurate, subgroup results were shown in pooled form if there were three or more studies for one subtype, otherwise, it was listed in an original form. Funnel plots were constructed and Begg’s and Egger’s tests were performed to assess the publication bias (*p* ≤ 0.10 was considered to be significant).

We analyzed the dose-response relationship using first-order, and second-order, and three-order fractional polynomial regression of the inverse variance-weighted data to estimate a curve of best fit. Best-fit curves were selected using decreased deviance compared with the reference model^48^. Comparisons of curves to determine best fit were done using a chi-square distribution. The average values within the blood TCDD categories were specified as the midpoint for bounded ranges, and 0.75 times the higher bound for the lowest (unbounded) range, and 1.25 times the lower bound for the highest (unbounded) range. RRs or SMRs (the ratio of observed to expected cancer deaths multiplied by 100) was the response measure used in these studies. All analyses were conducted using Stata software (version 12.0; StatCorp, College Station, TX, USA).

## Results

### Study characteristics and data quality

**Figure 1:**
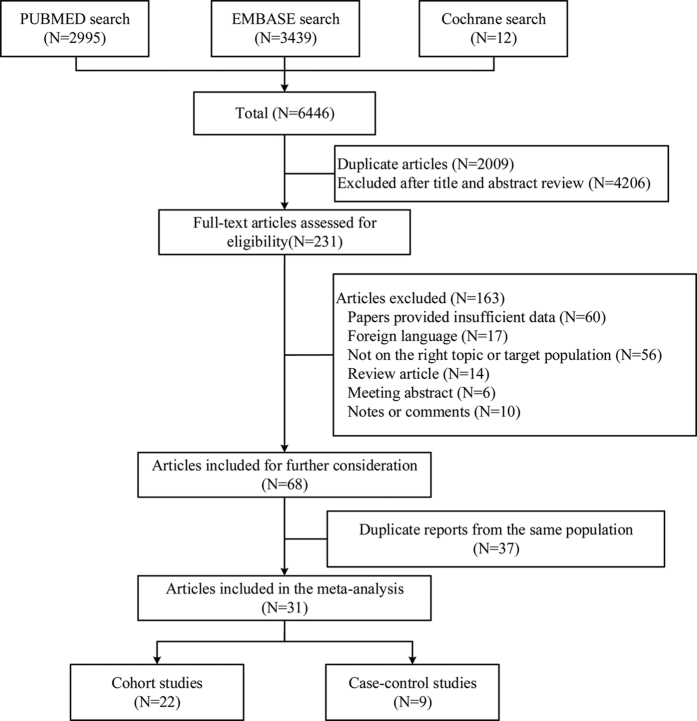
Flow diagram of study selection process.

After searching PUBMED, EMBASE and Cochrane library, 6446 articles were identified. 4437 articles were assessed after removing 2009 duplicate papers. Review of titles and abstracts resulted in exclusion of 4206 articles. For the remaining 231 articles, 163 were excluded for the following reasons: insufficient data (n = 60), foreign languages (n = 17), not on the right topic or targeted population (the outcomes of these studies were not cancer incidence or mortality, or the study interests were not dioxin) (n = 56), review articles (n = 14), meeting abstracts (n = 6), letters or comments (n = 10). 68 studies were included for further consideration and then 37 duplicate reports^49^,^50^,^51^,^52^,^53^,^54^,^55^,^56^,^57^,^58^,^59^,^60^,^61^,^62^,^63^,^64^,^65^,^66^,^67^,^68^,^69^,^70^,^71^,^72^,^73^,^74^,^75^,^76^,^77^,^78^,^79^,^80^,^81^,^82^,^83^,^84^,^85^ from the same population were excluded. The detailed study selection methods for the same population are shown in Supplementary Table 1. Finally, a total of 31 studies^10^,^11^,^12^,^13^,^14^,^15^,^16^,^17^,^18^,^19^,^20^,^21^,^22^,^23^,^24^,^25^,^26^,^27^,^28^,^29^,^30^,^31^,^32^,^33^,^34^,^35^,^36^,^37^,^38^,^39^,^40^ were included for the meta-analysis, including 22 cohort studies and 9 case-control studies. There were different TCDD exposure ways as follow: occupational exposure, non-occupational exposure, industrial accidents, and soldiers exposed to herbicides used in Vietnam War. The reference categories also varied among different studies, some adopted the non-exposed population to calculate SIRs or SMRs (external reference), and others adopted the lowest exposure categories (internal reference). We pooled the RRs or SMRs of high-exposed versus non-exposed categories for external reference, and highest versus lowest categories for the internal reference. Of note, all the included case-control studies only provided data on specific cancer types but no combined data on all cancer, and these studies were only pooled for the subgroup analysis but not for the all cancer analysis in order to ensure the accuracy of the results. The selection process is shown in Fig. 1, and the characteristics of the included studies are shown in Table 1. The exposure level and adjustment for confounders of included studies are shown in Supplementary Table 2.

Among the included studies, ten^13^,^20^,^22^,^25^,^31^,^32^,^34^,^38^,^39^,^40^ assessed the association between external exposure level of TCDD and cancer incidence. Eleven^10^,^11^,^12^,^15^,^16^,^17^,^18^,^20^,^21^,^29^,^30^ evaluated the association between external exposure level of TCDD and cancer mortality. For blood and adipose tissue level of TCDD, seven^14^,^19^,^26^,^33^,^35^,^36^,^37^ assessed cancer incidence and seven^14^,^16^,^23^,^24^,^27^,^28^,^29^ evaluated cancer mortality. Ott *et al*.^14^ reported the association between blood level of TCDD and both cancer incidence and mortality. Read *et al*.^20^ reported the association between external exposure of TCDD and both cancer incidence and mortality. Steenland *et al*.^16^ and Manuwald *et al*.^29^ reported the association between both external exposure and blood level of TCDD and cancer mortality. The results of quality assessment were shown in the Supplementary Table 3. The scores of most studies ranged from seven to nine (except for two studies got six points), which indicated the high quality of included studies and enhanced the reliability of the analysis. The PRISM checklist and flow diagram were shown in Supplementary Tables 4 and 5, respectively.

### External exposure of TCDD and cancer incidence and mortality

**Figure 2:**
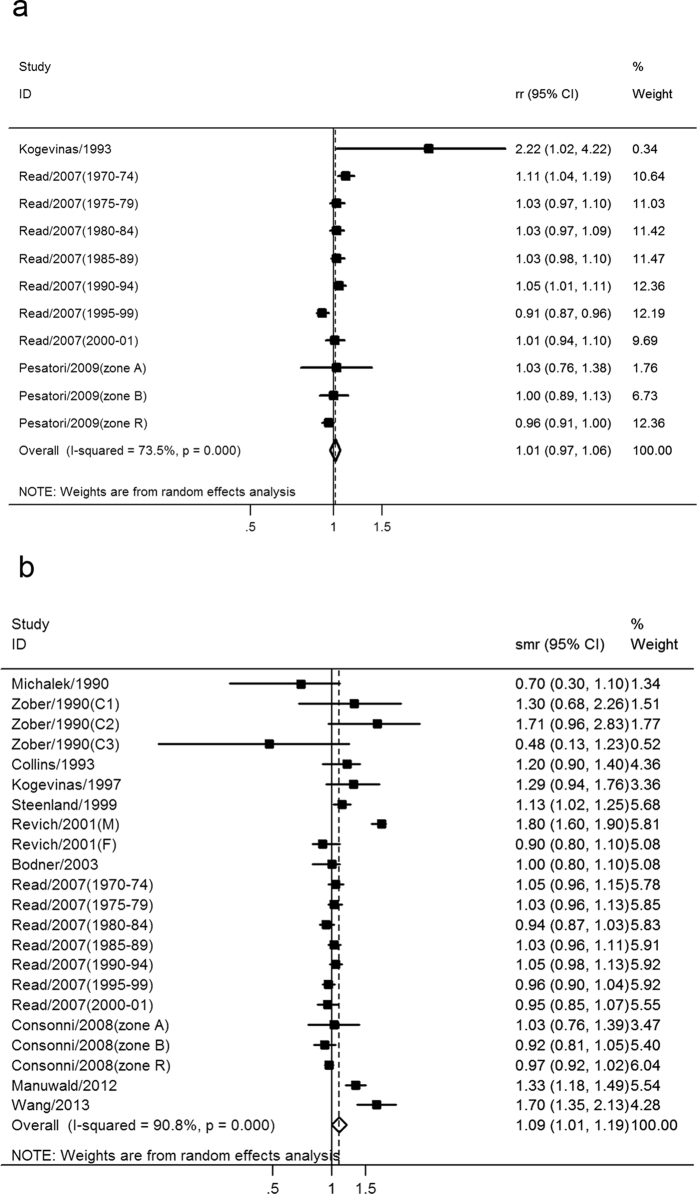
Meta-analysis of the association between external exposure level of TCDD and (**a**) all cancer incidence and (**b**) all cancer mortality.

Ten studies involving 18,969 cancer cases and 3,155,159 participants assessed the association between external exposure of TCDD and cancer incidence, including five cohort studies and five case-control studies. The pooled RR of all cancer incidence of TCDD exposure level was 1.01 (95% CI: 0.97–1.06), indicating no significant association (Fig. 2a). There was significant heterogeneity across the included studies (I^2^ = 73.5%, *p* < 0.001), as shown in Fig. 2a. Subgroup analysis was conducted according to cancer subtype, as shown in Table 2. The pooled RRs of different cancer types were all not significant, including breast cancer, Hodgkin’s lymphoma, lymphatic leukemia, non-Hodgkin’s lymphoma, and soft-tissue sarcoma. The results of subgroup analysis suggested the heterogeneity may be caused by special cancer types. Sensitivity analysis was also conducted to further explain the source of heterogeneity according to quality assessment results. After exclusion of the study^13^ of the lowest score (six points), the pooled RR was 1.01(95% CI: 0.97–1.05), while the heterogeneity was not significantly changed (from I^2^ = 73.5% to I^2^ = 72.7%).

Eleven studies involving 9,122 cancer deaths and 691,326 participants assessed the association between external exposure of TCDD and cancer mortality. The pooled SMR of all cancer mortality of TCDD exposure level was 1.09 (95% CI: 1.01–1.19), indicating a significant positive association (Fig. 2b). There was significant heterogeneity across the included studies (I^2^ = 90.8%, *p* < 0.001), as shown in Fig. 2b. Subgroup analyses for the association between external exposure of TCDD and cancer mortality were conducted according to cancer types and TCDD exposure ways, as shown in Table 2. The pooled SMRs of cancer mortality were significant in esophagus cancer (pooled SMR = 1.52, 95% CI: 1.09–2.13), larynx cancer (pooled SMR = 2.2, 95% CI: 1.61–3.02), kidney cancer (pooled SMR = 1.39, 95% CI: 1.08–1.78), non-Hodgkin’s lymphoma (pooled SMR = 1.18, 95% CI: 1.01–1.37), myeloma (pooled SMR = 1.49, 95% CI: 1.03–2.15), soft-tissue sarcoma (pooled SMR = 1.60, 95% CI: 1.15–2.23), and occupational exposed population (pooled SMR = 1.25, 95% CI: 1.07–1.46). Subgroup analyses suggested that heterogeneity was partly influenced by cancer type and TCDD exposure way (Table 2). To further explore the potential impact of within-study heterogeneity, we also conducted sensitivity analyses according to the quality assessment results. After excluded the study^10^ of the lowest score (six points), the pooled SMR was 1.10 (95% CI: 1.01–1.20), while the heterogeneity was not significantly changed (from I^2^ = 90.8% to I^2^ = 91.2%). The efficiency of the current sensitivity analysis was not able to provide evidence to further explain the source of heterogeneity.

### Blood level of TCDD and cancer incidence and mortality

**Figure 3:**
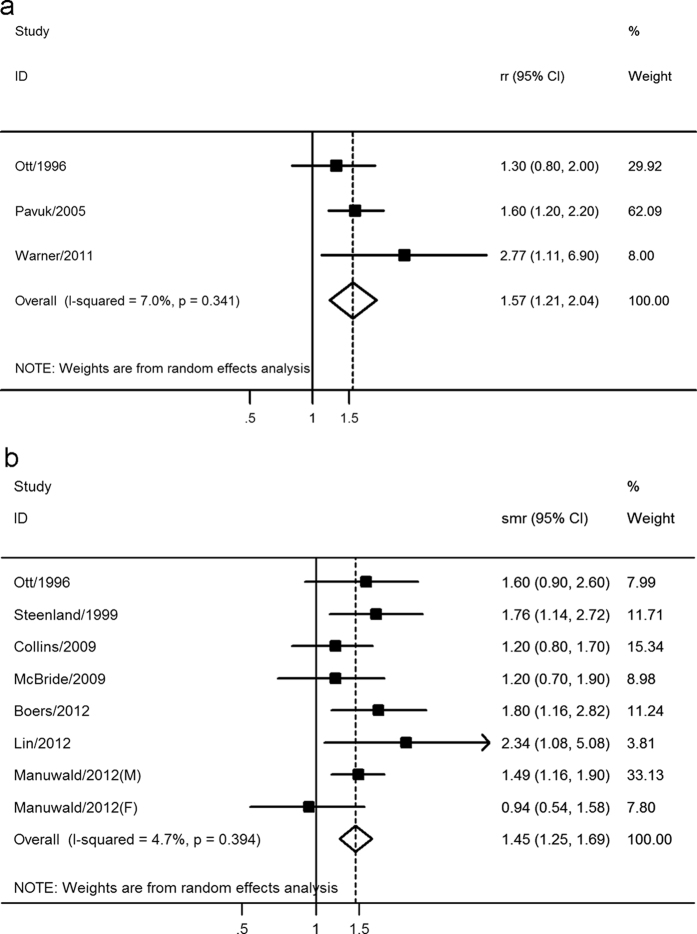
Meta-analysis of the association between blood level of TCDD and (**a**) all cancer incidence and (**b**) all cancer mortality.

Seven studies comprising 837 cancer cases and 3,446 participants evaluated the association between blood of TCDD and cancer incidence, including three cohort studies and four case-control studies. The pooled RR of all cancer incidence for the highest versus lowest categories of TCDD exposure level was 1.57 (95% CI: 1.21–2.04), indicating a positive significant association (Fig. 3a). The I^2^ and *p* value for heterogeneity across the included studies were 7.0% and 0.341 respectively, as shown in Fig. 3a. Subgroup analysis was not conducted due to the limited data.

Seven studies involving 997 cancer deaths and 13,793 participants assessed the association between blood level of TCDD and cancer mortality. The pooled SMR of all cancer mortality for the highest versus lowest categories of TCDD exposure level was 1.45 (95% CI: 1.25–1.69), indicating a significant positive association (Fig. 3b). There was no significant heterogeneity across the included studies (I^2^ = 4.7%, *p* = 0.394), as shown in Fig. 3b. Subgroup analysis was conducted according to cancer type, exposure way and reference category. Two studies assessed the association between blood level of TCDD and non-Hodgkin’s lymphoma, and the SMRs (95% CI) were 4.50 (1.20–11.50) and 1.36 (1.06–1.74), respectively. The results suggested a significant positive association, which was consistent with the results of higher exposure level of TCDD. However, the results should be treated cautiously considering the relatively small sample size (n = 11), and more studies were needed to validate it. The subgroup analyses also indicated that it was all significant for occupational exposed and non-occupational exposed population, and for external and internal reference category, which further verified the stability of the results.

**Figure 4:**
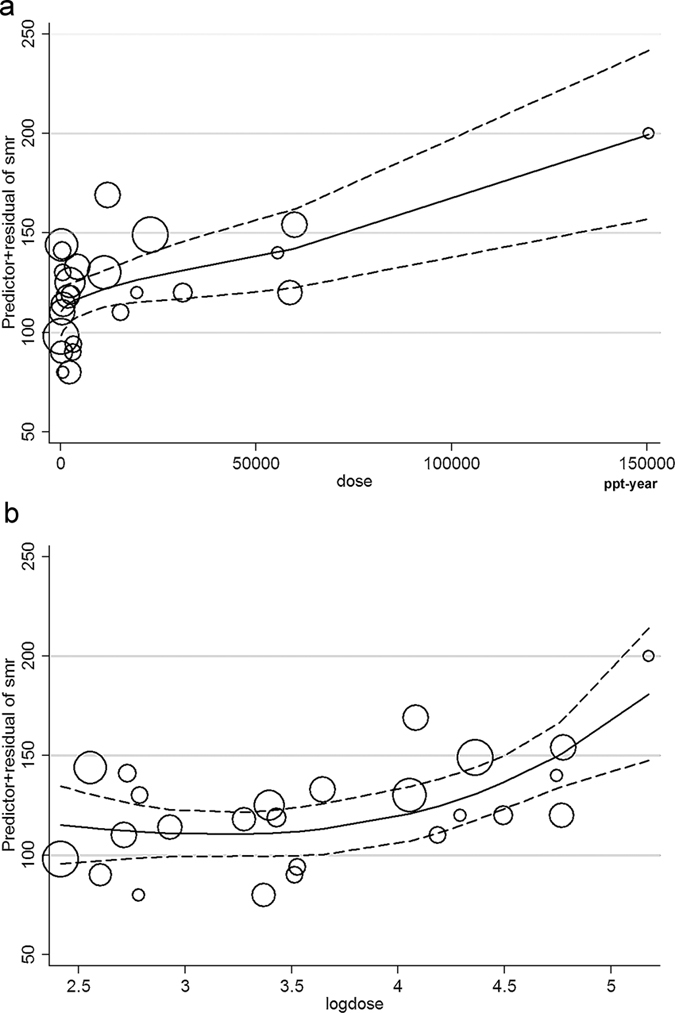
Dose-response analysis of the association between blood level of TCDD and all cancer mortality. (**a**) Dose relationship between blood TCDD level and all cancer SMR. (**b**) Log dose relationship between blood TCDD level and all cancer SMR. The solid line represents SMRs and the dotted line represents 95% confidence intervals.

Dose-response analysis was conducted based on five studies^14^,^16^,^23^,^24^,^29^ according to the model of two-order fractional polynomial regression. RRs or SMRs using the low exposure group as the reference group were not appropriate for the dose-response analysis, which needs the RRs or SMRs relative to the normal background uncontaminated by occupational dioxin exposure^43^. Crump *et al*.^43^ conducted a dose-response analysis in 2003 with only three studies. The raw data of Ott *et al*.^14^ and Steenland *et al*.^16^,^77^ was obtained by personal communication by the authors^43^, thus we used these data extracted from Crump *et al*.^43^ to improve the validity of our analysis. We adopted Manuwald *et al*.’s study^29^ rather than Flesch-Janys *et al*.’s^63^ for the Hamburg cohort since the former had a longer follow-up time. Cumulative serum lipid concentration (CSLC, ppt-years) was selected as the exposure metric to relate to risk, and the second-order fractional polynomial regression plot indicated a positive correlation between blood TCDD level and all cancer SMR, as shown in Fig. 4a. After log transformation of TCDD dose, the curve showed a non-linear increasing trend (Fig. 4b). The size of the circles in Fig. 4 represented the study sample size. The SMRs remained below 114.02 for serum TEQ dose from 316.23 ppt-years to 5141.62 ppt-years. For the TEQ dose of 1000, 10000, 100000 ppt-years, the SMRs with 95% CIs were 110.67(99.09–122.26), 119.82(105.79–133.23) and 167.68(141.77–194.21), respectively. With SMRs increased from 114.02 to 124.02, the TEQ dose increased form 5141.62 ppt-years to 14883.33 ppt-years.

### Publication bias

Begg’s funnel plots and Egger’s linear regression test indicated no evidence of publication bias in the present study (TCDD external exposure and cancer incidence P_Begg_ = 0.755 and P_Egger_ = 0.245, and mortality P_Begg_ = 0.150 and P_Egger_ = 0.521; blood level of TCDD and cancer incidence P_Begg_ = 1.000 and P_Egger_ = 0.620, and mortality P_Begg_ = 0.711 and P_Egger_ = 0.834). The funnel plots were shown in Supplementary Figures 1 to 4.

## Discussion

The current meta-analysis summarized the results of twenty-two cohort studies and nine case-control studies, including ten on external exposure level of TCDD and cancer incidence, eleven on external exposure level and cancer mortality, seven on blood level of TCDD and cancer incidence, and seven on blood level of TCDD and cancer mortality. The results indicated that higher external exposure level of TCDD was significantly associated with all cancer mortality but not all cancer incidence. For external exposure studies, the dioxin exposure ways, exposure quantification methods, reference categories, exposure level and adjustment for potential confounders differed greatly among included studies, which could cause heterogeneity and these results should be taken cautiously. Besides, there was a significantly positive association between higher blood level of TCDD and both all cancer incidence and mortality. The subgroup analysis for TCDD exposure mortality reported significant results for esophagus cancer, larynx cancer, kidney cancer, non-Hodgkin’s lymphoma, myeloma, soft-tissue sarcoma and occupational exposed population. However, the IARC’s review suggested that the evidence for specific cancers was strongest for lung cancer, soft-tissue sarcoma and non-Hodgkin’s lymphoma^3^. The IARC’s review listed the related publications, while they didn’t distinguish the duplicated studies based on the same population and didn’t provided quantitatively pooled results. Thus, the results of the current study may provide relatively more detailed indications on specific cancer types. Interestingly, the subgroup analysis also suggested consistence for increased mortality ratio of non-Hodgkin’s lymphoma in both higher external exposure and blood level of TCDD, which may provide evidence on the precise carcinogenic potency of TCDD from an epidemiological point of view. The dose-response analysis showed an increasing trend of SMR with higher blood TEQ dose. For the TEQ dose of 1000, 10000, 100000 ppt-year, the SMRs were 110.67, 119.82 and 167.68, respectively.

The present meta-analysis provided epidemiological evidence for the carcinogenic potency of TCDD and the subgroup analysis showed specific cancer sites. Importantly, the consistent results for non-Hodgkin’s lymphoma mortality of both external exposure and blood level of TCDD may indicate its specific effect on hematopoietic system. Although the sample size was relative small in the blood level of TCDD and non-Hodgkin’s lymphoma mortality subgroup analysis, the results of the included two studies were both significant, independently. The SMRs and sample size of non-Hodgkin’s lymphoma by Collins *et al*.^23^ and Boers *et al*.^27^ were 4.50 (1.2–11.5, n = 4) and 1.36 (1.06–1.74, n = 7), respectively, which suggested possibility that the association may be especially significant for non-Hodgkin’s lymphoma. It has been reported by Hardell *et al*.^86^ that exposure to phenoxy acids, chlorophenols and organic solvents may be a causative factor in malignant lymphoma as early as 1981. And based on decades of research, it has been realized that, exposure to dioxins, in particular TCDD could induce chloracne^87^, and WHO has also classified it as a human carcinogen^3^. In consideration of the extensive sources, widespread trend and the strong toxicity of TCDD, the present results have considerable epidemiological and public health importance for humans. However its carcinogenic potential to humans and the mechanisms are not clearly demonstrated. It’s commonly believed that AhR activation accounted for most biological properties of dioxins, including various physiological and developmental processes, tumor promotion, thymic involution, craniofacial anomalies, skin disorders and alterations in the endocrine, immunological and reproductive systems^50^,^88^. Furthermore, TCDD may also up-regulate drug-metabolizing enzymes, thus increasing the presence of highly reactive intermediates that form during metabolic activation and/or transformation of several key hormones^3^. Animal experiment also suggested that intraperitoneal injection of TCDD could cause increased incidence of lymphomas in male and female mice^89^.

Determining the sources of heterogeneity is an important goal of meta-analysis. The heterogeneity of our study mainly existed in external exposure level of TCDD and all cancer incidence (I^2^ = 73.5%, p < 0.001) and mortality (I^2^ = 90.8%, p < 0.001). Subgroup analyses suggested that cancer subtype and dioxin exposure way can partially explain heterogeneity across the studies. Sensitivity analysis was also conducted according to the quality assessment results, while the efficiency was not able to provide evidence to further explain the source of heterogeneity. However, the heterogeneity caused by different TCDD exposure ways, quantification methods, reference categories (internal or external), lag time, background exposure levels and adjustment for confounders couldn’t be fully quantified due to the limitation of individual participant data. The future research should pay more attention to the unity of survey methods and the standardization of the exposure reference category to control heterogeneity.

Our study has several strengths. First, we adopted the external exposure and blood level of TCDD to thoroughly assess the association between TCDD and cancer incidence and mortality. Second, subgroup analyses and dose-response analyses were applied, which further strengthened the conclusions and emphasized the TCDD effects on some specific cancer sites. Although the 2012 IARC monographs^3^ evaluated the evidence in humans for the carcinogenicity of TCDD and made a list of cohort studies, these issues were not systematically reviewed and quantified by a meta-analysis. Thus, the current meta-analysis fill in gaps in the IARC deficiencies on this issue and it’s of considerable interest and public health importance. In addition, no publication bias was observed, indicating that the pooled results should be unbiased.

However, the current analysis is restricted by several limitations. First, the number of studies involved in blood level of TCDD and all cancer incidence was relatively small, and thus some of the subgroup analyses were difficult to conduct. Second, in the dose-response analysis, the normal background uncontaminated by occupational dioxin exposure was different, and only McBride *et al*.^24^ study provided the New Zealand background level of 3.9 ppt. We didn’t add the background exposure level to our analysis for the limitation of original data. Third, the Steenland *et al*.^16^ used a 15-year lag time, whereas no lag was used in other cohorts. Although the Crump *et al*.’s analysis^43^ inferred that results based on cumulative exposure lagged 15 years should not differ greatly from those based on unlagged exposure, this could cause inaccuracy and heterogeneity. Thus, the individual participant data meta-analysis is needed to enhance future analysis. Fourth, the subgroup analysis for blood level of TCDD and all cancer mortality was limited in digestive system, respiratory system, lung cancer, prostate cancer and non-Hodgkin’s lymphoma. More studies with precise data of different cancer types are warranted to support the effects of TCDD on other cancers.

In conclusion, our findings suggest that external exposure and blood level of TCDD were both significantly associated with all cancer mortality. Higher external exposure of TCDD may significantly increase the mortality rate of esophagus cancer, larynx cancer, kidney cancer, non-Hodgkin’s lymphoma, myeloma, soft-tissue sarcoma and occupational exposure population. Of note, such relationship may be especially significant for non-Hodgkin’s lymphoma.

## Additional Information

**How to cite this article**: Xu, J. *et al*. Association between dioxin and cancer incidence and mortality: a meta-analysis. *Sci. Rep*. **6**, 38012; doi: 10.1038/srep38012 (2016).

**Publisher's note:** Springer Nature remains neutral with regard to jurisdictional claims in published maps and institutional affiliations.

## Supplementary Material

Supplementary Information

## Figures and Tables

**Table 1:** Characteristics of included studies.

No.	Study	Country/cohort	Time period	Exposure way	Exposure assessment	Reference category	Cancer types	Gender	No. of cancer cases/cohort or controls	Study quality	Age (years)	Duplicated reports
Cohort studies												
	Exposure incidence											
1	Kogevinas^13^	part of IARC*	1955–1988	occupational	job records, company records and detailed company exposure questionnaires	External: SIR and SMR	all cancer, breast cancer	F	29/701	6	N/A	
2	Read^20^	New Zealand	1970–2001	non-occupational	individual’s recorded Territorial Authority for usual place of residence at death or cancer registration	External: New Plymouth population	all cancer, lymphocytic leukemia, Hodgkin’s disease, Non-Hodgkin’s lymphoma, soft tissue sarcoma	F/M	8013/375583	8	N/A	
3	Viel^22^	French#	1990–1999	non-occupational	modelled ground-level concentrations	External: Isere population	non-Hodgkin’s lymphoma	F/M	3974/2487274	8	mean 61.49 ± 16.21	
4	Pesatori^25^	Italy, Seveso	1977–1996	industrial accident	measurements of TCDD soil levels	External: surrounding non-contaminated territory including 11 municipalities	All cancer, Esophagus, stomach, colon, rectum, liver, biliary tract, pancreas, lung, pleura, soft tissue sarcoma, melanoma, skin, breast, genito-urinary tract, ovary, prostate, testis, bladder, kidney, brain, thyroid, Hodgkin’s disease, non-Hodgkin’s lymphoma, leukemia	F/M	2122/218761	8	0–74	Pesatori^72^, Bertazzi^52^, Pesatori^73^
5	Danjou^31^	French, E3N cohort	1993–2008	non-occupational	diet history questionnaire	Internal: the lowest category	breast cancer	F	3465/63830	9	mean 52.73 ± 6.58	
	Exposure mortality											
1	Michalek^10^	USA, vietnam veterans-AFSH	1982–1987	Vietnam war	physical Examination, Ranch Hands veterans	External: the comparison veterans	all cancer	M	12/2294	6	48.5	
2	Zober^11^	Germany-BASF Aktiengesellschaft	1953–1987	industrial accident	company records	External: national mortality rate	all cancer, buccal cavity and pharynx, esophagus, stomach, colon, rectum, larynx, lung, bone, skin, prostate, bladder, leukemia	F/M	23/247	8	mean 63.4	
3	Collins^12^	USA, West Virginia, Monsanto company	1949–1987	industrial accident	work records and Internal Revenue Service Form	External: local population mortality rate	all cancer, stomach, colorectal, liver and biliary, respiratory system, bone, skin, prostate, bladder, lymphatic and hematopoietic, soft-tissue sarcoma	M	102/754	7	N/A	
4	Kogevinas^15^	IARC, 36cohorts$	1939–1992	occupational	job records, company records and detailed company exposure questionnaires	External: SIR and SMR	all cancer, buccal cavity and pharynx, esophagus, stomach, colon, rectum, liver and biliary, pancreas, peritoneum, nose and nasal sinuses, larynx, lung, bone, skin, prostate, kidney, testis, bladder, breast, cervix, endometrium and uterus, leukemia, Hodgkin’s disease, non-Hodgkin’s lymphoma, myeloma, brain, soft tissue sarcoma, thyroid	F/M	710/21863	7	N/A	Saracci^76^, Kogevinas^67^, Bueno de Mesquita^58^, Kogevinas^13^, Vena^79^, Kogevinas^85^
5	Steenland^16^	USA, NIOSH	1942–1993	occupational	job records, job-exposure matrix and blood sample test	External (US non-exposed people) and Internal (the lowest category)	all cancer, esophagus, stomach, colon, rectum, liver and biliary, pancreas, peritoneum, larynx, lung, prostate, kidney, bladder, lymphatic and hematopoietic, leukemia, Hodgkin’s disease, non-Hodgkin’s lymphoma, myeloma, brain and nervous system, connective tissue and soft tissue	M	377/5172	7	N/A	Fingerhut^61^, Steenland^78^, Salvan^75^
6	Revich^17^	Russia	1983–1997	non-occupational	food and soil concentration test	External: death rate in Samara Region	all cancer, intestine, stomach, colon, rectum, larynx, lung, bone, soft-tissue, breast, cervix, urinary organs, leukemia, lymphomas	F/M	803/-	8	N/A	
7	Bodner^18^	USA-Michigan, Dow chemical company	1940–1994	occupational	job records and exposure score	External: other area workers with background exposure to dioxin	all cancer, lung, soft-tissue sarcoma, non-Hodgkin’s lymphoma	M	168/2187	7	N/A	Cook^60^, Ott^70^, Bond^57^, Ramlow^74^
8	Read^20^	New Zealand	1970–2001	non-occupational	individual’s recorded Territorial Authority for usual place of residence at death or cancer registration	External: New Plymouth population	all cancer, lymphocytic leukemia, Hodgkin’s disease, Non-Hodgkin’s lymphoma, soft tissue sarcoma	F/M	4235/375583	8	N/A	
9	Consonni^21^	Italy, Seveso	1976–2001	industrial accident	measurements of TCDD soil levels	External: surrounding non-contaminated territory including 11 municipalities	all cancer, stomach, colon, rectum, liver, biliary tract, pancreas, lung, soft tissue sarcoma, melanoma, breast, genito-urinary tract, ovary, prostate, bladder, kidney, brain, Hodgkin’s disease, non-Hodgkin’s lymphoma, leukemia	F/M	2278/278108	8	0–74	Bertazzi^56^, Bertazzi^55^, Bertazzi^54^, Bertazzi^53^, Baccarelli^50^
10	Manuwald^29^	Germany, Hamburg, Boehringer Ingelheim	1952–2007	occupational	company records and blood or fat tissue samples	External: Hamburg population	all cancer, hypo pharynx, digestive organs, esophagus, stomach, colon, rectum, pancreas, larynx, lung, pleura, breast, prostate, kidney, bladder, hematopoietic system, non-Hodgkin’s lymphoma	F/M	291/1589	7	N/A	Manz^68^
11	Wang^30^	China	1980–2005	occupational	air sample concentration test	External: Chinese national mortality rates	all cancer, lung, liver, gastric	F/M	121/3529	7	N/A	
	Blood incidence											
1	Ott^14^	Germany, Ludwigshafen	1959–1992	occupational	questionnaire and blood sample	External: West Germany population	all cancer, buccal cavity, digestive organs, stomach, colorectal, liver, gall bladder or bile duct, respiratory system, lung, prostate, bladder or kidney, lymphatic or hematopoietic tissue, skin	M	47/243	7	N/A	
2	Pavuk^19^	USA, vietnam veterans	1982–2003	Vietnam war	physical examination and blood sample	Internal: the lowest category	all cancer, all SEER sites, digestive system, respiratory system, melanoma, basal or squamous cell, prostate	M	402/1482	8	mean 63.7	Ketchum^66^, Akhtar^49^, Pavuk^71^, Michalek^69^
3	Warner^26^	Italy, Seveso, SWHS cohort	I:1976–1996, II:1997–2009	industrial accident	interview, physical examination and blood sample	Internal: the lowest category	all cancer, breast cancer	F	66/981	9	0–40	Warner^80^
	Blood mortality											
1	Ott^14^	Germany, Ludwigshafen	1959–1992	occupational	questionnaire and blood sample	External: West Germany population	all cancer, digestive organs, respiratory system, prostate, bladder or kidney, lymphatic or hematopoietic tissue	M	31/243	7	N/A	Zober^11^
2	Steenland^16^	USA, NIOSH	1942–1993	occupational	job records, job-exposure matrix and blood sample test	External (US non-exposed people) and Internal (the lowest category)	all cancer, lung cancer	M	256/5172	8	N/A	Steenland^77^, Cheng^59^
3	Collins^23^	USA, Michigan	1937–1980	occupational	job records and blood sample test	External (US population) and Internal (the lowest category)	all cancer, lung, prostate, kidney, non-Hodgkin’s lymphomas	M	94/773	8	mean 31.1	
4	McBride^24^	New Zealand	1969–2004	occupational	job records and blood sample test	External (New Zealand population) and internal (the lowest category)	all cancer, digestive organs, lung, soft-tissue sarcoma, lymphatic and hematopoietic tissue, non-Hodgkin’s lymphoma	F/M	61/1599	8	mean 52.9	
5	Boers^27^	Netherlands, Dutch cohort	1955–2006	occupational	blood sample test and predictive model	Internal (background exposure level as reference)	all cancer, digestive organs, stomach, pancreas, respiratory system, lung, skin, genital and urinary cancer, prostate, bladder, kidney, lymphatic and hematopoietic cancer, non-Hodgkin’s lymphoma, leukemia	M	192/2056	8	N/A	Heederik^64^, Hooiveld^65^
6	Lin^28^	USA, NHANES	1999–2006	non-occupational	blood sample test	Internal (the lowest category)	all cancer	F/M	72/2361	8	>40	
7	Manuwald^29^	Germany, Hamburg	1952–2007	occupational	company records and blood or fat tissue samples	External: Hamburg population	all cancer, digestive organs, respiratory system, breast cancer	F/M	291/1589	7	N/A	Flesch-Janys^62^, Bencher/51, Flesch-Janys^63^
Case-control studies												
	Exposure incidence											
1	Hardell^32^	Sweden	1970–1986	non-occupational	structured questionnaire and work history	Internal (unexposed)	soft-tissue sarcoma	M	434/948	6	25–80	Hardell^81^, Eriksson^82^, Hardell^83^, Eriksson^84^
2	Floret^34^	France, Besançon	1980–1995	non-occupational	modeled ground-level according to meteorological conditions	Internal (the lowest category)	non-Hodgkin’s lymphoma	F/M	222/2220	6	median 66	
3	Zambon^38^	Italy, Venice	1990–1996	non-occupational	survey of the incinerators and industrial sources of airborne dioxin	Internal (the lowest category)	sarcoma	F/M	172/405	6	N/A	
4	Viel^39^	France, Besançon	1996–2002	non-occupational	modeled ground-level according to meteorological conditions	Internal (the lowest category)	breast cancer	F	434/2170	6	>20	
5	Villeneuve^40^	Eight European countries‖	1995–1997	occupational	structured questionnaire and work history	Internal (the lowest category)	male breast cancer	M	104/1901	6	35–70	
	Blood and adipose tissue incidence											
1	Hardell^33^	Sweden	1994–1997	non-occupational	adipose tissue sample test	Internal (the lowest category)	non-Hodgkin’s lymphoma	NA	33/39	7	NA	
2	Tuomisto^35^	Finland	1997–1999	non-occupational	fat sample test and questionnaire	Internal (the lowest category)	soft-tissue sarcoma	F/M	110/227	7	15.0–91.1	
3	De Roos^36^	US, NCI; SEER, the parent study	1998–2000	non-occupational	blood sample test	Internal (the lowest category)	non-Hodgkin’s lymphoma	F/M	100/100	5	20–74	
4	Reynolds^37^	US	mid-1990s	non-occupational	adipose tissue sample test and questionnaire	Internal (the lowest category)	breast cancer	F	79/52	6	mainly 40–59	

IARC: The International Agency for Research on Cancer.

E3N: Etude Epidémiolog ique auprès de femmes de la Mutuelle Générale de l’Education Nationale.

AFSH: air force health study.

NIOSH: National Institute for Occupational Safety and Health.

SWHS: the Seveso Women’s Health Study.

NHANES: National Health and Nutrition Examination Survey.

F: female, M: male, N/A: not available.

Study quality was judged on the basis of the Newcastle-Ottawa Scale (1–9 stars).

^*^Austria, Denmark, Finland, Italy, Netherlands, New Zealand, and Sweden.

^#^Four administrative departments, Isère, Bas-Rhin, Haut-Rhin and Tarn.

^$^Australia, Austria, Canada, Denmark, Finland, Italy, the Netherlands, New Zealand, Sweden, UK, Germany, USA.

^‖^Denmark, France, Germany, Italy, Sweden, Latvia, Portugal and Spain.

**Table 2:** Subgroup analyses of the association between TCDD and cancer incidence and mortality.

Categories	Classification	Study number	No. of cases	RR or SMR (95% CI)	Heterogeneity		Study
					I^2^	p	
Exposure incidence							
cancer type	breast cancer	3	3768	0.99(0.93–1.06)	9.30%	0.356	
	Hodgkin’s lymphoma	2	49	1.13(0.83–1.54)	—	—	Read^20^
			26	1.42(0.93–2.18)	—	—	Pesatori^25^
	lymphatic leukemia	2	104	1.35(0.93–1.97)	—	—	Read^20^
			13	0.83(0.46–1.48)	—	—	Pesatori^25^
	non-Hodgkin’s lymphoma	4	4263	1.09(0.92–1.30)	65.80%	0.001	
	soft-tissue sarcoma	4	105	1.37(0.97–1.93)	48.70%	0.041	
Exposure mortality							
cancer type	buccal cavity and pharynx	2	22	1.30(0.82–1.97)	—	—	Kogevinas^15^
			11	2.17(1.08–3.87)*	—	—	Manuwald^29^
	esophagus	3	44	1.52(1.09–2.13)*	9.10%	0.333	
	stomach	7	433	1.02(0.82–1.27)	68.10%	0.001	
	colorectal	7	453	1.05(0.94–1.19)	20.10%	0.214	
	colon	5	298	0.97(0.86–1.09)	0.00%	0.532	
	rectum	5	154	1.18(0.97–1.44)	25.10%	0.238	
	liver and biliary	5	212	1.01(0.79–1.30)	0.00%	0.046	
	pancreas	4	139	0.93(0.78–1.11)	0.00%	0.719	
	peritoneum	2	5	2.19(0.45–6.41)	—	—	Steenland^16^
			3	1.23(0.40–2.80)	—	—	Kogevinas^15^
	larynx	4	45	2.20(1.61–3.02)*	0.00%	0.563	
	trachea/lung	8	1190	1.21(0.89–1.65)	95.20%	<0.001	
	prostate	5	172	1.14(0.97–1.34)	0.00%	0.830	
	kidney	4	90	1.39(1.08–1.78)*	16.60%	0.309	
	bladder	5	117	1.73(0.95–3.18)	89.00%	<0.001	
	Hodgkin’s disease	4	43	1.35(0.97–1.88)	0.00%	0.895	
	non-Hodgkin’s lymphoma	6	239	1.18(1.01–1.37)*	20.10%	0.235	
	myeloma	3	50	1.49(1.03–2.15)*	24.80%	0.256	
	leukemia	5	156	1.14(0.96–1.35)	0.00%	0.464	
	skin	2	9	0.89(0.36–2.18)	—	—	Kogevinas^15^
			3	0.85(0.49–1.48)	—	—	Consonni^21^
	brain nervous system	3	57	0.91(0.69–1.20)	0.00%	0.418	
	bone	2	2	5.00(0.60–18.1)	—	—	Collins^12^
			3	1.08(0.22–3.14)	—	—	Kogevinas^15^
	soft-tissue sarcoma	6	46	1.60(1.15–2.23)*	0.00%	0.550	
	breast	4	234	1.27(0.78–2.06)	87.80%	<0.001	
	endometrium and uterus	2	3	3.41(0.70–9.96)	—	—	Kogevinas^15^
43			0.99(0.44–2.24)	—	—	Consonni^21^	
exposure way	non-occupational	2	803	1.28(0.65–2.52)	—	—	Revich^17^
			4235	1.00(0.97–1.04)	—	—	Read^20^
	occupational	5	1667	1.25(1.07–1.46)*	78.30%	0.001	
	industrial accident	3	2405	1.02(0.91–1.14)	44.80%	0.093	
	Vietnam war	1	12	0.70(0.30–1.10)	—	—	Michalek^10^
Serum mortality							
cancer type	digestive organs	4	82	1.22(0.88–1.69)	44.00%	0.147	
	respiratory system	3	82	1.25(0.86–1.81)	57.50%	0.095	
	lung	4	74	0.99(0.86–1.15)	0.00%	0.450	
	prostate	2	4	1.40(0.40–3.60)	—	—	Collins^23^
			14	1.08(0.79–1.49)	—	—	Boers^27^
	non-Hodgkin’s lymphoma	2	4	4.50(1.20–11.50)*	—	—	Collins^23^
			7	1.36(1.06–1.74)*	—	—	Boers^27^
exposure way	non-occupational	1	72	2.34(1.08–5.08)*	—	—	Lin^28^
	occupational	4	925	1.43(1.23–1.66)*	0.00%	0.442	
reference category	external	5	733	1.39(1.18–1.63)*	0.00%	0.458	
	internal	2	192	1.80(1.16–2.82)*	—	—	Boers^27^
			72	2.34(1.08–5.08)*	—	—	Lin^28^

— Could not be calculated.

^*^Significant association was indicated, statistical z test: p < 0.05.
