# Corrigendum: Association between dioxin and cancer incidence and mortality: a meta-analysis

**DOI:** 10.1038/srep41665

**Published:** 2017-02-10

**Authors:** Jinming Xu, Yao Ye, Fang Huang, Hanwen Chen, Han Wu, Jian Huang, Jian Hu, Dajing Xia, Yihua Wu

Scientific Reports 6: Article number: 38012; 10.1038/srep38012 published online: 11 29 2016; updated: 02 10 2017.

The Acknowledgements section in this Article is incomplete.

‘The work was supported by grants from the National Natural Science Foundation of China (Grant Nos 81400371). The funders had no role in study design, data collection and analysis, decision to publish, or preparation of the manuscript. The authors wish to thank Dr. Xinqiang Zhu and Dr. Jun Zhang (Department of Toxicology, Zhejiang University School of Public Health) for valuable discussion and suggestion’.

Should read:

‘The work was supported by grants from the National Natural Science Foundation of China (Grant Nos 81302455 and 31471297). The funders had no role in study design, data collection and analysis, decision to publish, or preparation of the manuscript. The authors wish to thank Dr. Xinqiang Zhu and Dr. Jun Zhang (Department of Toxicology, Zhejiang University School of Public Health) for valuable discussion and suggestion’.

