# Supplementary material for: Association between dioxin and cancer incidence and mortality: a meta-analysis
Source: Sci Rep. 2016 Nov 29;6:38012. doi: 10.1038/srep38012 (PMC5126552; doi:10.1038/srep38012)
Supplement: Supplementary Information [file srep38012-s1.doc]

**Association between dioxin and cancer incidence and mortality: a meta-analysis**

Jinming Xu^1#^, Yao Ye^1#^, Fang Huang^1^, Hanwen Chen^1^, Han Wu^2^, Jian Huang^3^, Jian Hu^4^, Dajing Xia^1^*, Yihua Wu^1^*

^1^Department of Toxicology, Zhejiang University School of Public Health, Hangzhou, 310058 China; ^2^Second Affiliated Hospital, Zhejiang University School of Medicine, Hangzhou 310009, China; ^3^Department of Oncology, Second Affiliated Hospital, Zhejiang University School of Medicine, Hangzhou 310009, China; ^4^Department of Thoracic Surgery, The first Affiliated Hospital, Zhejiang University School of Medicine, Hangzhou 310003, China

**Supplementary Files**

**Supplementary Table 1.** Criteria to select the studies conducted in the same population

| **Study** | **Duplicated reports** | **Selection Reason** |
| --- | --- | --- |
| **Cohort studies** | | |
| Pesatori/2009[^25^](#_ENREF_25) | Pesatori/1992[^72^](#_ENREF_72), Bertazzi/1993[^52^](#_ENREF_52), Pesatori/1993[^73^](#_ENREF_73) | most recent publication |
| Kogevinas/1997[^15^](#_ENREF_15) | Saracci/1991[^76^](#_ENREF_76), Kogevinas/1992[^67^](#_ENREF_67), Bueno de Mesquita/1993[^58^](#_ENREF_58), Kogevinas/1993[^13^](#_ENREF_13), Vena/1998[^79^](#_ENREF_79), Kogevinas/1995[^85^](#_ENREF_85)^#^ | most detailed information |
| Steenland/1999[^16^](#_ENREF_16) | Fingerhut/1991[^61^](#_ENREF_61), Steenland/1992[^78^](#_ENREF_78), Salvan/2001[^75^](#_ENREF_75) | most detailed information |
| Bodner/2003[^18^](#_ENREF_18) | Cook/1986[^60^](#_ENREF_60), Ott/1987[^70^](#_ENREF_70), Bond/1989[^57^](#_ENREF_57), Ramlow/1996[^74^](#_ENREF_74) | most recent publication |
| Consonni/2008[^21^](#_ENREF_21) | Bertazzi/1989[^56^](#_ENREF_56), Bertazzi/1992[^55^](#_ENREF_55), Bertazzi/1997[^54^](#_ENREF_54), Bertazzi/2001[^53^](#_ENREF_53), Baccarelli/2004[^50^](#_ENREF_50) | most recent publication |
| Manuwald/2012[^29^](#_ENREF_29) | Manz/1991[^68^](#_ENREF_68) | most recent publication |
| Pavuk/2005[^19^](#_ENREF_19) | Ketchum/1999[^66^](#_ENREF_66), Akhtar/2004[^49^](#_ENREF_49), Pavuk/2006[^71^](#_ENREF_71), Michalek/2008[^69^](#_ENREF_69) | most detailed information |
| Warner/2011[^26^](#_ENREF_26) | Warner/2002[^80^](#_ENREF_80) | more recent publication |
| Ott/1996[^14^](#_ENREF_14) | Zober/1990[^11^](#_ENREF_11) | more recent publication |
| Steenland/1999[^16^](#_ENREF_16) | Steenland/2001[^77^](#_ENREF_77), Cheng/2006[^59^](#_ENREF_59) | most detailed information |
| Boers/2012[^27^](#_ENREF_27) | Heederik/1998[^64^](#_ENREF_64), Hooiveld/1998[^65^](#_ENREF_65) | most recent publication |
| Manuwald/2012[^29^](#_ENREF_29) | Flesch-Janys/1995[^62^](#_ENREF_62), Bencher/1998[^51^](#_ENREF_51), Flesch-Janys/1998[^63^](#_ENREF_63) | most recent publication |
| **Case-control studies** | | |
| Hardell/1993[^32^](#_ENREF_32) | Hardell/1979[^81^](#_ENREF_81), Eriksson/1981[^82^](#_ENREF_82), Hardell/1988[^83^](#_ENREF_83), Eriksson/1990[^84^](#_ENREF_84) | most recent publication^*^ |

The criteria priority of study selection was established according to (1) whether the detailed information of different cancer subtypes or dioxin exposure level was provided, or studies with a larger sample size and (2) the publication time. As shown in the above table, if the selection reason was “most recent publication”, the other criteria (detailed data information and the sample size) of the selected study were equal or better than the other studies assessed the same population.

^#^: This was a case-control study which was based on the same population by the same author’s cohort study in 1997.

^*^: The most recent publication was a “meta-analysis” of the four previous studies. The precise exposure materials described by the four duplicated reports were phenoxyacetic acid and chlorophenols, while the expressions were “dioxin” and “TCDD” in the most recent study.

**Supplementary Table 2.** Additional information for characteristics of the included studies.

|  | **No.** | **Study** | **TCDD exposure level** | **adjustment for potential confounders** |
| --- | --- | --- | --- | --- |
| **Cohort studies** | | | | |
|  | **exposure incidence** | | | |
|  | 1 | Kogevinas/1993 | probable  unlikely | - |
|  | 2 | Read/2007 | site of manufacturing plant | census undercount |
|  | 3 | Viel/2008 | cumulative ground-level dioxin concentration | five possible confounders (no specific description) |
|  | 4 | Pesatori/2009 | Mean soil TCDD  Zone A 15.5-580.4 ug/m^2^  Zone B 1.7-4.3 ug/m^2^  Zone R 0.9-1.4 ug/m^2^  Reference NA | gender, age category, and period |
|  | 5 | Danjou/2015 | Dietary dioxin exposure  ＜0.98 pg/kg body weight (BW)/d  0.98-1.23 pg/kg body weight (BW)/d  1.23-1.52 pg/kg body weight (BW)/d  ≥1.52 pg/kg body weight (BW)/d | age, height, body mass index, energy intake, education, physical activity, smoking status, menopausal status combined with use of menopausal hormone treatment, alcohol intake, age at menarche, use of oral contraceptives, use of progestin, age at menopause, age at first full-term pregnancy and number of live births, breastfeeding, family history of breast cancer, history of personal benign breast disease and mammography |
|  | **exposure mortality** | | | |
|  | 1 | Michalek/1990 | Vietnam war, no specific description | Age, calendar year in 5-year intervals, rank(flyer or non-flyer) |
|  | 2 | Zober/1990 | Years science first exposure, chloracne and partial blood sample  0-9  10-19  20+ | - |
|  | 3 | Collins/1993 | By development of chloracne or by their holding a job with potential exposure | - |
|  | 4 | Kogevinas/1997 | Ten cohorts and each had a different mean exposure level | Country, age, sex, calendar period, time science first exposure, duration of exposure and employment status |
|  | 5 | Steenland/1999 | cumulative  0-39 ppt-years  39-224 ppt-years  224-791 ppt-years  791-2120 ppt-years  2120-6140 ppt-years  6140-15800 ppt-years  >15800 ppt-years | the concentration factor over time |
|  | 6 | Revich/2001 | Dioxin in soil (141.3 ng TEQ/kg)  Dioxin in vegetables (0.07 pg TEQ/kg in carrots, 0.021 pg TEQ/kg in potatoes)  Drinking water (less 2 pg/litter)  Human milk of 40 women (23.2 pg TEQ/g/fat)  Female workers’ blood sample (80.5 pg TEQ/lipid) | - |
|  | 7 | Bodner/2003 | No specific data, only categories provided  Background  Very low  Low  Moderate  High  Very high | - |
|  | 8 | Read/2007 | site of manufacturing plant | census undercount |
|  | 9 | Consonni/2008 | Mean soil TCDD and partial serum sample  Zone A 15.5-580.4 ug/m^2^  Zone B 1.7-4.3 ug/m^2^  Zone R 0.9-1.4 ug/m^2^  Reference NA | presence at the accident, gender, period (1976–1981, 1982–1986, 1987–1991, 1992–1996, and 1997–2001), age (<1, 1–4 years, then 5-year categories until age 84 years, and >85 years), and time since first exposure (‘‘latency,’’ 0–4, 5–9, 10–14, 15–19, and >20 years) |
|  | 10 | Manuwald/2012 | Total cumulative exposure for each individual was calculated as the sum of the cumulative exposures in each of the workplaces where the worker had been employed. For each workplace, cumulative exposure was calculated as the product of total time spent in a workplace and the workplace specific exposure estimated previously. | - |
|  | 11 | Wang/2013 | Air samples (pg/Nm^3^)  S1: 0.07±0.02  S2: 0.05±0.00  S3: 0.07±0.04  S4: 0.15±0.10  S5: 0.31±0.14  S6: 0.02±0.01  Dust samples(pg/g)  S1: 1.80±0.05  S2: 0.68±0.08  S3: 1.23±0.09  S4: 0.55±0.08  S5: 5.38±0.73  S6: 2.98±0.33 | age, gender, smoking status, crystalline silica exposure, duration of employment and education levels |
|  | **blood incidence** | | | |
|  | 1 | Ott/1996 | ＜0.1 μg/kg  0.1-0.99μg/kg  ≥1μg/kg | age、sex、calendar period specific cancer incidence rate |
|  | 2 | Pavuk/2005 | 0.4-2.6pg/g Lipid 2.6-3.8pg/g Lipid 3.8-5.2pg/g Lipid 5.2-54.8pg/g Lipid | Adjusted for military occupation, year of birth, the number of years served in SEA, BMI at the qualifying tour, the relative change in BMI from the qualifying tour to the TCDD measurement, baseline smoking history (pack-years), and baseline drinking history (drink-years) |
|  | 3 | Warner/2011 | ≤20 ppt 20.1-47.0 ppt 47.1-135.0 ppt ＞135 ppt | marital status、age at explosion |
|  | **blood mortality** | | | |
|  | 1 | Ott/1996 | ＜0.1 μg/kg  0.1-0.99μg/kg  ≥1μg/kg | Age, sex, calendar period specific cancer incidence rate |
|  | 2 | Steenland/1999 | cumulative  0-39 ppt-years  39-224 ppt-years  224-791 ppt-years  791-2120 ppt-years  2120-6140 ppt-years  6140-15800 ppt-years  >15800 ppt-years | the concentration factor over time |
|  | 3 | Collins/2009 | 0.01-0.69 ppt-years 0.70-3.99 ppt-years 4.00-113.37 ppt-years | - |
|  | 4 | McBride/2009 | cumulative  0-68.3 ppt-mo 68.4-475.0 ppt-mo 475.1-2085.7 ppt-mo ＞2085.8 ppt-mo | Age, sex, hire year, birth year |
|  | 5 | Boers/2012 | ≤0.4ppt 0.4-4.1ppt 4.1-20.1ppt ≥20.1ppt | age |
|  | 6 | Lin/2012 | 0-13.3 pgTEQ/g lipid 13.3-27.9 pgTEQ/g lipid ＞27.9 pgTEQ/g lipid | attained age, gender, body mass index, race/ethnicity, cigarette smoking, and alcohol consumption |
|  | 7 | Manuwald/2012 | men  0-13.1 ppt 13.1-77.4 ppt 77.1-334.5 ppt ≥334.5 ppt women 0 ppt 0-19.5 ppt 19.5-78.3 ppt ＞78.3 ppt | - |
| **Case-control studies** | | | | |
|  | **exposure incidence** | | | |
|  | 1 | Hardell/1993 | unexposed  exposed < 1 year  exposed ≥ 1 year | - |
|  | 2 | Floret/2003 | very low (modeled ground-level dioxin concentration<0.0001 pg/m^3^ zone)  low (modeled ground-level dioxin concentration 0.0001–0.0002 pg/m^3^ zone)  intermediate (modeled ground-level dioxin concentration 0.0002–0.0004 pg/m^3^ zone)  high (modeled ground-level dioxin concentration 0.0004–0.0016 pg/m^3^ zone) | age, gender, educational level, workers in labor force, employment, single woman as head of household, owner-occupied houses, number of persons per dwelling, single-family houses. |
|  | 3 | Zambon/2007 | Average exposure (fgr/m^3^)  <4 fgr/m^3^  4-6 fgr/m^3^  ≥6 fgr/m^3^ | - |
|  | 4 | Viel/2008 | very low (modeled ground-level dioxin concentration<0.0001 pg/m^3^ zone)  low (modeled ground-level dioxin concentration 0.0001–0.0002 pg/m^3^ zone)  intermediate (modeled ground-level dioxin concentration 0.0002–0.0004 pg/m^3^ zone)  high (modeled ground-level dioxin concentration 0.0004–0.0016 pg/m^3^ zone) | age, gender, educational level, workers in labor force, employment, single woman as head of household, owner-occupied houses, number of persons per dwelling, single-family houses. |
|  | 5 | Villeneuve/2010 | cumulative exposure score  not exposure  <median  ≥median | age, country, alcohol consumption, body mass index and exposures to other environmental estrogens |
|  | **blood and adipose tissue incidence** | | | |
|  | 1 | Hardell/2001 | ≤3.2 pg/g lipid  >3.2 pg/g lipid | age, sex, BMI |
|  | 2 | Tuomisto/2004 | WHO-TEq, ng/kg in fat  5 categoties | sex |
|  | 3 | De Roos/2005 | quartiles(mol/g lipid)  ≤0.746 mol/g lipid  0.746-1.083 mol/g lipid  1.083-1.888 mol/g lipid  >1.888 mol/g lipid | sex, study site, birth date, and date of blood draw. |
|  | 4 | Reynolds/2005 | ≤2.1 pg/g  2.2-3.8 pg/g  >3.8 pg/g | age and race |

-: not available

**Supplementary Table 3**. Quality assessment of the included studies according to NEWCASTLE-OTTAWA standard.

|  |  | **Study** | **Q1** | **Q2** | **Q3** | **Q4** | **Q5** | **Q6** | **Q7** | **Q8** | **Quality** |
| --- | --- | --- | --- | --- | --- | --- | --- | --- | --- | --- | --- |
| **Cohort studies** | | | | | | | | | | | |
|  | **Exposure incidence** | | | | | | | | | | |
|  | 1 | Kogevinas/1993 | c | b | a | a | ab | d | a | b | 6 |
|  | 2 | Read/2007 | a | b | a | a | ab | b | a | b | 8 |
|  | 3 | Viel/2008 | a | a | a | b | ab | b | a | b | 8 |
|  | 4 | Pesatori/2009 | a | b | a | a | ab | b | a | b | 8 |
|  | 5 | Danjou/2015 | a | a | b | a | ab | a | a | b | 9 |
|  | **Exposure mortality** | | | | | | | | | | |
|  | 1 | Michalek/1990 | c | b | a | a | a | b | a | b | 6 |
|  | 2 | Zober/1990 | a | b | a | a | ab | b | a | b | 8 |
|  | 3 | Collins/1993 | a | b | a | a | a | b | a | b | 7 |
|  | 4 | Kogevinas/1997 | c | b | a | a | ab | b | a | b | 7 |
|  | 5 | Steenland/1999 | c | b | a | a | ab | b | a | b | 7 |
|  | 6 | Revich/2001 | a | b | a | a | ab | b | a | b | 8 |
|  | 7 | Bodner/2003 | c | b | a | a | ab | b | a | b | 7 |
|  | 8 | Consonni/2008 | a | b | a | a | ab | b | a | b | 8 |
|  | 9 | Read/2007 | a | b | a | a | ab | b | a | b | 8 |
|  | 10 | Manuwald/2012 | c | b | a | a | ab | b | a | b | 7 |
|  | 11 | Wang/2013 | c | b | a | a | ab | a | a | b | 7 |
|  | **Blood incidence** | | | | | | | | | | |
|  | 1 | Ott/1996 | c | b | a | a | ab | a | a | b | 7 |
|  | 2 | Pavuk/2005 | c | a | a | a | ab | a | a | b | 8 |
|  | 3 | Warner/2011 | a | a | a | a | ab | b | a | b | 9 |
|  | **Blood mortality** | | | | | | | | | | |
|  | 1 | Ott/1996 | c | b | a | a | ab | a | a | b | 7 |
|  | 2 | Steenland/1999 | c | a | a | a | ab | b | a | b | 8 |
|  | 3 | Collins/2009 | c | a | a | a | ab | b | a | b | 8 |
|  | 4 | McBride/2009 | c | a | a | a | ab | b | a | b | 8 |
|  | 5 | Boers/2012 | c | a | a | a | ab | b | a | b | 8 |
|  | 6 | Lin/2012 | a | a | a | a | ab | b | b | b | 8 |
|  | 7 | Manuwald/2012 | c | b | a | a | ab | b | a | b | 7 |
| **Case-control studies** | | | | | | | | | | | |
|  | **Exposure incidence** | | | | | | | | | | |
|  | 1 | Hardell/1993 | a | a | a | b | ab | c | a | b | 6 |
|  | 2 | Floret/2003 | b | a | a | b | ab | c | a | a | 6 |
|  | 3 | Zambon/2007 | b | a | a | a | ab | c | a | c | 6 |
|  | 4 | Viel/2008 | b | a | a | b | ab | c | a | a | 6 |
|  | 5 | Villeneuve/2010 | a | a | b | b | ab | c | a | a | 6 |
|  | **Blood and adipose tissue incidence** | | | | | | | | | | |
|  | 1 | Hardell/2001 | c | a | a | a | ab | c | a | a | 7 |
|  | 2 | Tuomisto/2004 | a | a | a | b | ab | c | a | a | 7 |
|  | 3 | De Roos/2005 | b | a | c | b | ab | c | a | a | 5 |
|  | 4 | Reynolds/2005 | a | a | c | b | ab | c | a | a | 6 |

**Quality assessment of cohort studies**

Q1: Representativeness of the exposed cohort

a) truly representative of the average population in the community * b) somewhat representative of the average population in the community

c) selected group of users d) no description of the derivation of the cohort

Q2:Selection of the non exposed cohort

a) drawn from the same community as the exposed cohort *b) drawn from a different source

c) no description of the derivation of the non-exposed cohort

Q3: Ascertainment of exposure

a) secure record * b) structured interview *

c) written self-report d) no description

Q4: Demonstration that outcome of interest was not present at start of study

a) yes * b) no

Q5: Comparability of cohorts on the basis of the design or analysis

a) study controls for age b) study controls for any additional factor Outcome

Q6: Assessment of outcome

a) independent blind assessment * b) record linkage *

c) self-report d) no description

Q7: Was follow-up long enough for outcomes to occur

a) yes * b) no

Q8: Adequacy of follow up of cohorts

a) complete follow up - all subjects accounted for *

b) subjects lost to follow up unlikely to introduce bias - small number lost > 70 % follow up, or description provided of those lost

c) follow up rate < 70% and no description of those lost

d) no statement

**Quality assessment of case-control studies**

Q1: Is the case definition adequate?

a) yes, with independent validation * b) yes, eg record linkage or based on self-reports c) no description

Q2: Representativeness of the cases

a) consecutive or obviously representative series of cases *b) potential for selection biases or not stated

Q3: Selection of Controls

a) community controls * b) hospital controls c) no description

Q4: Definition of Controls

a) no history of disease (endpoint) * b) no description of source

Q5: Comparability of cases and controls on the basis of the design or analysis

a) study controls for age * b) study controls for any additional factor

Q6: Ascertainment of exposure

a) secure record * b) structured interview where blind to case/control status *

c) interview not blinded to case/control status d) written self-report or medical record only

e) no description

Q7: Same method of ascertainment for cases and controls

a) yes *b) no

Q8: Non-Response rate

a) same rate for both groups * b) non respondents described c) rate different and no designation

**Supplementary Table 4.** The PRISMA checklist.

| **Section/topic** | **#** | **Checklist item** | **Reported on page #** |
| --- | --- | --- | --- |
| **TITLE** | | |  |
| Title | 1 | Identify the report as a systematic review, meta-analysis, or both. | 1 |
| **ABSTRACT** | | |  |
| Structured summary | 2 | Provide a structured summary including, as applicable: background; objectives; data sources; study eligibility criteria, participants, and interventions; study appraisal and synthesis methods; results; limitations; conclusions and implications of key findings; systematic review registration number. | 2 |
| **INTRODUCTION** | | |  |
| Rationale | 3 | Describe the rationale for the review in the context of what is already known. | 3-4 |
| Objectives | 4 | Provide an explicit statement of questions being addressed with reference to participants, interventions, comparisons, outcomes, and study design (PICOS). | 3-4 |
| **METHODS** | | |  |
| Protocol and registration | 5 | Indicate if a review protocol exists, if and where it can be accessed (e.g., Web address), and, if available, provide registration information including registration number. | 4-7 |
| Eligibility criteria | 6 | Specify study characteristics (e.g., PICOS, length of follow-up) and report characteristics (e.g., years considered, language, publication status) used as criteria for eligibility, giving rationale. | 4-5 |
| Information sources | 7 | Describe all information sources (e.g., databases with dates of coverage, contact with study authors to identify additional studies) in the search and date last searched. | 4 |
| Search | 8 | Present full electronic search strategy for at least one database, including any limits used, such that it could be repeated. | 4 |
| Study selection | 9 | State the process for selecting studies (i.e., screening, eligibility, included in systematic review, and, if applicable, included in the meta-analysis). | 5 |
| Data collection process | 10 | Describe method of data extraction from reports (e.g., piloted forms, independently, in duplicate) and any processes for obtaining and confirming data from investigators. | 5-6 |
| Data items | 11 | List and define all variables for which data were sought (e.g., PICOS, funding sources) and any assumptions and simplifications made. | 5-6 |
| Risk of bias in individual studies | 12 | Describe methods used for assessing risk of bias of individual studies (including specification of whether this was done at the study or outcome level), and how this information is to be used in any data synthesis. | 6-7 |
| Summary measures | 13 | State the principal summary measures (e.g., risk ratio, difference in means). | 6-7 |
| Synthesis of results | 14 | Describe the methods of handling data and combining results of studies, if done, including measures of consistency (e.g., I^2^) for each meta-analysis. | 6-7 |

Page 1 of 2

| **Section/topic** | **#** | **Checklist item** | **Reported on page #** |
| --- | --- | --- | --- |
| Risk of bias across studies | 15 | Specify any assessment of risk of bias that may affect the cumulative evidence (e.g., publication bias, selective reporting within studies). | 6 |
| Additional analyses | 16 | Describe methods of additional analyses (e.g., sensitivity or subgroup analyses, meta-regression), if done, indicating which were pre-specified. | 6-7 |
| **RESULTS** | | |  |
| Study selection | 17 | Give numbers of studies screened, assessed for eligibility, and included in the review, with reasons for exclusions at each stage, ideally with a flow diagram. | 7 |
| Study characteristics | 18 | For each study, present characteristics for which data were extracted (e.g., study size, PICOS, follow-up period) and provide the citations. | 7-8 |
| Risk of bias within studies | 19 | Present data on risk of bias of each study and, if available, any outcome level assessment (see item 12). | 9-12 |
| Results of individual studies | 20 | For all outcomes considered (benefits or harms), present, for each study: (a) simple summary data for each intervention group (b) effect estimates and confidence intervals, ideally with a forest plot. | 9-12 |
| Synthesis of results | 21 | Present results of each meta-analysis done, including confidence intervals and measures of consistency. | 9-12 |
| Risk of bias across studies | 22 | Present results of any assessment of risk of bias across studies (see Item 15). | 12 |
| Additional analysis | 23 | Give results of additional analyses, if done (e.g., sensitivity or subgroup analyses, meta-regression [see Item 16]). | 9-12 |
| **DISCUSSION** | | |  |
| Summary of evidence | 24 | Summarize the main findings including the strength of evidence for each main outcome; consider their relevance to key groups (e.g., healthcare providers, users, and policy makers). | 12-17 |
| Limitations | 25 | Discuss limitations at study and outcome level (e.g., risk of bias), and at review-level (e.g., incomplete retrieval of identified research, reporting bias). | 16 |
| Conclusions | 26 | Provide a general interpretation of the results in the context of other evidence, and implications for future research. | 16-17 |
| **FUNDING** | | |  |
| Funding | 27 | Describe sources of funding for the systematic review and other support (e.g., supply of data); role of funders for the systematic review. | 17 |

*From:*  Moher D, Liberati A, Tetzlaff J, Altman DG, The PRISMA Group (2009). Preferred Reporting Items for Systematic Reviews and Meta-Analyses: The PRISMA Statement. PLoS Med 6(6): e1000097. doi:10.1371/journal.pmed1000097


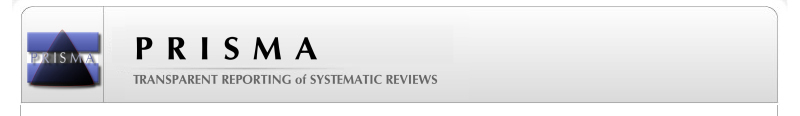
**PRISMA 2009 Flow Diagram**

**Screening**

**Included**

**Eligibility**

**Identification**

Records identified through database searching
(n = 6446 )

Additional records identified through other sources
(n = 0 )

Records after duplicates removed
(n = 4437 )

Records screened
(n = 4437 )

Records excluded
(n = 4206)

Full-text articles assessed for eligibility
(n = 231 )

Full-text articles excluded, with reasons
(n = 163)

Studies included in qualitative synthesis
(n = 68)

Studies included in quantitative synthesis (meta-analysis)
(n = 31)

**Supplementary Table 4.** The PRISMA flow diagram.

*From:*  Moher D, Liberati A, Tetzlaff J, Altman DG, The PRISMA Group (2009). *P*referred *R*eporting *I*tems for *S*ystematic Reviews and *M*eta-*A*nalyses: The PRISMA Statement. PLoS Med 6(6): e1000097. doi:10.1371/journal.pmed1000097

**For more information, visit** [**www.prisma-statement.org**](http://www.consort-statement.org/)**.**

**Supplementary Figure 1**. Funnel plot of TCDD external exposure and cancer incidence.

**Supplementary Figure 2**. Funnel plot of TCDD external exposure and cancer mortality.

**Supplementary Figure 3**. Funnel plot of serum TCDD level and cancer incidence.

**Supplementary Figure 4**. Funnel plot of serum TCDD level and cancer mortality.
